# Early renal function trajectories, cytomegalovirus serostatus and long-term graft outcomes in kidney transplant recipients

**DOI:** 10.1186/s12882-021-02285-2

**Published:** 2021-03-20

**Authors:** Jonathan P. Law, Richard Borrows, David McNulty, Adnan Sharif, Charles J. Ferro

**Affiliations:** 1 Institute of Cardiovascular Sciences, College of Medical and Dental Sciences, Edgbaston, Birmingham, B15 2TT UK; 2 Department of Renal Medicine, University Hospitals Birmingham NHS Foundation Trust, Birmingham, B15 2GW UK; 3 Department of Medical Informatics, University Hospitals Birmingham NHS Foundation Trust, Birmingham, B15 2GW UK

**Keywords:** Allograft function, Bayesian, Cytomegalovirus serostatus, Estimated glomerular filtration rate, Kidney transplantation

## Abstract

**Background:**

Improved recognition of factors influencing graft survival has led to better short-term kidney transplant outcomes. However, efforts to prevent long-term graft decline and improve graft survival have seen more modest improvements. The adoption of electronic health records has enabled better recording and identification of donor-recipient factors through the use of modern statistical techniques. We have previously shown in a prevalent renal transplant population that episodes of rapid deterioration are associated with graft loss.

**Methods:**

Estimated glomerular filtration rates (eGFR) between 3 and 27 months after transplantation were collected from 310 kidney transplant recipients. We utilised a Bayesian approach to estimate the most likely eGFR trajectory as a smooth curve from an average of 10,000 Monte Carlo samples. The probability of having an episode of rapid deterioration (decline greater than 5 ml/min/1.73 m^2^ per year in any 1-month period) was calculated. Graft loss and mortality data was collected over a median follow-up period of 8 years. Factors associated with having an episode of rapid deterioration and associations with long-term graft loss were explored.

**Results:**

In multivariable Cox Proportional Hazard analysis, a probability greater than 0.8 of rapid deterioration was associated with long-term death-censored graft loss (Hazard ratio 2.17; 95% Confidence intervals [CI] 1.04–4.55). In separate multivariable logistic regression models, cytomegalovirus (CMV) serostatus donor positive to recipient positive (Odds ratio [OR] 3.82; 95%CI 1.63–8.97), CMV donor positive (OR 2.06; 95%CI 1.15–3.68), and CMV recipient positive (OR 2.03; 95%CI 1.14–3.60) were associated with having a greater than 0.8 probability of an episode of rapid deterioration.

**Conclusions:**

Early episodes of rapid deterioration are associated with long-term death-censored graft loss and are associated with cytomegalovirus seropositivity. Further study is required to better manage these potentially modifiable risks factors and improve long-term graft survival.

**Supplementary Information:**

The online version contains supplementary material available at 10.1186/s12882-021-02285-2.

## Background

The last three decades have seen a remarkable improvement in renal transplant survival, a product of technological advances in surgical technique and medical care. In particular, the availability of novel and potent immunosuppressive drugs has been paralleled by strategies to reduce immunosuppression-related toxicity and opportunistic infections [1]. In addition to improving graft survival, maintenance of transplant graft function, or glomerular filtration rate, is another key strategy to minimise the complications associated with advanced chronic kidney disease (CKD) and improve patient survival [1, 2]. Deterioration in renal transplant function remains a significant issue, and ranks amongst the top four causes of end-stage renal disease (ESRD) in the United States [3]*.* Kidney transplant recipients rank transplant survival the most important outcome [4]. Although early outcomes after kidney transplantation have improved markedly over the last couple of decades, improvements in long-term outcomes have been much more modest [1, 5]. The major factors for graft loss are well established and usually reflect patient and donor characteristics at the time of transplantation (e.g. donor age, immunological mismatch) that are essentially non-modifiable [6]. However, potentially modifiable risk factors including cytomegalovirus (CMV) serostatus/mismatch are emerging as possible therapeutic targets [7].

The increasing adoption of Electronic Health Record (EHR) systems in recent years [8] has resulted in the accumulation of a massive amount of structured data on patients and their disease deterioration. It has been argued that that the quality of care of CKD and kidney transplant recipients could be improved by effective utilisation of EHR [9]. However, most studies examining change in renal function have been fairly simplistic and assume a linear decline [10, 11]. Furthermore, prior survival analyses have tended to rely on single baseline timepoint measurements, without consideration for fluctuations of the measurement over time and how these impact on the outcome during the observation period. Alternatively, repeated measurements of parameters, for example estimated glomerular filtration rate (eGFR), allows quantification of the variability and provide a better estimation of the true trajectory over time. The trajectory and the nature of the variability can then be used to explore associations with outcomes of interest [12].

Although there has been an increased interest in examining renal function trajectories in CKD [13, 14], there is little work in kidney transplantation [15]. Work from CKD groups and our own in renal transplant recipients [16] suggest that most patients do not experience linear renal function. Instead, many experience periods of non-progression and episodes of rapid decline. We have previously shown in a prevalent kidney transplant population that episodes of rapid deterioration of renal function were frequent and more likely in patients who subsequently lost their grafts [16]. Whether this is true in incident patients is unknown. Understanding the trajectories of kidney allograft (dys) function, especially in the early post-transplantation period, is key to understanding mechanisms behind graft dysfunction and subsequent failure, and the implementation of preventative strategies.

The purpose of this study was threefold:

1. Investigate the probability of episodes of rapid deterioration of renal function in an early period (3–27 months) post-transplantation in an unselected, incident population of kidney allograft recipients.
2. Evaluate baseline factors associated with an episode of rapid deterioration of eGFR during the early (3–27 months) period post-transplantation.
3. Probe whether episodes of rapid deterioration of renal function during the early (3–27 months) period post-transplantation are associated with subsequent graft loss in an extended follow-up period.

## Methods

### Study population

We used a comprehensive database, created by data linkage between a number of EHR, of all adult patients aged 18 or over with ESRD who received a kidney-only transplant at our centre between 21st January 2007 and 31st December 2013. Data for every study participant were extracted from the Department of Health Informatics database, with manual record linkage to additional EHR: graft survival was acquired from the UK Transplant Registry held by NHS Blood and Transplant; patient survival data were obtained from the Office for National Statistics. Patients were included if they remained under our follow-up and were not repatriated to their original referring hospital post-transplantation, and were alive with a working graft 27 months after transplantation. Patients with missing donor and/or recipient CMV data were excluded. Patients were classified based on CMV serostatus: donor and recipient seronegative (D−/R−), donor seronegative and recipient seropositive (D−/R+), donor seropositive and recipient seronegative (D+/R−), and donor and recipient seropositive (D+/R+). Survival analysis was censored to event or 31st November 2018, whichever occurred first.

We utilised existing ethnicity classifications as obtained from EHR, which were cross-checked against UK Transplant Registry data. Ethnicity was classified into white, black, south Asian (also referred to as Indo-Asian) or other.

Determination of socioeconomic deprivation was based on the Index of Multiple Deprivation (IMD), a composite score encompassing multiple domains reflective of areas of socioeconomic deprivation. The IMD scores are divided into quintiles, 1 represents the most deprived and 5 represents the least deprived area.

### Immunosuppression and Cytomegalovirus prophylaxis protocol

All patients received the same immunosuppression in line with the SYMPHONY protocol over the study period. Induction therapy was with basiliximab 10 mg twice/day and methylprednisolone 500 mg on the day of transplantation. Maintenance therapy included tacrolimus (target 12-h trough level: 5−8 ng/L), mycophenolate mofetil (2 g/day with tapering to 1 g/day after 6 months), and prednisolone. Patients who were deemed high risk (D+/R−) received 3 months of valganciclovir post-transplant.

### Assessment of an episode of rapid deterioration in the estimated glomerular filtration rate trajectory

All eGFR values up to 27 months after transplantation were retrieved. Values in the first 3 months post-transplantation were excluded because of the intrinsic variability of renal function in the immediate post-transplantation period giving 24 months of values for analysis. We used a Bayesian smoothing technique to estimate each patient’s eGFR trajectory as a smooth curve in the observation [16]. This technique produces a smooth curve for each individual patient that reflects the more gradual, longer-term changes in eGFR values, rather than the more rapid, short-term changes because of clinical and biologic variation as well as other interference including measurement error. The smoothness of the curve was determined automatically by the data based on the prespecified model. For each individual patient, the Bayesian approach produced 10,000 Monte Carlo samples to approximate the posterior distribution of all modelling parameters, which led to 10,000 curves that quantified the uncertainty in the true trajectory given the variation of the data. Under the Bayesian approach we estimated the “most-likely” trajectory by the average of those 10,000 Monte Carlo curves. The Bayesian approach allows estimation of the probability that a patient’s trajectory had a feature of interest as a proportion of the 10,000 Monte Carlo curves that showed this feature. The estimated trajectory is a smooth curve, allowing its slope to be calculated month by month, accommodating a possible change in rate of deterioration over time.

We calculated the probability of a period of rapid deterioration of renal function as an eGFR trajectory having at least 1 month in which eGFR declined by at least 5 mL/min/1.73 m^2^ per year. This is the threshold conventionally used in CKD guidelines to define a rapid decline in renal function [17]. Patients were considered to have had at least 1 period of rapid deterioration if the probability was ≥0.8. Sensitivity analyses were conducted using probabilities ≥0.70 and 0.90.

### Statistical analysis

Continuous data are expressed as mean ± standard deviation for parametric data or median (25th–75th quartiles) for nonparametric data, and compared using Student *t*-test or Mann-Whitney U test respectively. Categorical data are presented as percentages and compared using the chi-square or Fisher’s exact tests.

Our outcomes of interest were having a probability of rapid deterioration of renal function ≥0.8 in the 2-year observation period (3–27 months post-transplant) and graft and patient survival during the follow-up period (27 months post-transplant until data censored). Graft failure was taken as the time from transplant to return to dialysis, graft nephrectomy, or repeat kidney transplant (whichever occurred first, with death data censored). Patient survival was defined as the time from transplant until death.

Logistic regression analysis was used to model the binary outcome of having a probability of rapid deterioration of renal function ≥0.8 in the observation period and Cox proportional hazards regression models were used for time-to-event outcome analysis for graft loss and all-cause mortality. For graft survival and all-cause mortality, Kaplan-Meier analyses were run with Mantel-Cox (log-rank) tests used to compare patient groups and to test the proportionality hazards assumption. Covariates included in the multivariable analyses were any with *P* < 0.10 in unadjusted analyses. Closely-correlated factors (e.g. CMV serostatus combinations, CMV D+ and CMV R+) were entered one at a time into ***separate*** multivariable models. Transplant outcomes were compared using D−/R− as reference. A separate model which included only recipients with CMV seropositive donors (D+/R− vs D+/R+; using D+/R− as a reference group) was analysed. Models were tested using backward and forward entry methods. Data was 100% complete for all covariates and outcomes of interest.

Analyses were performed using R 3.5.1 (R Foundation, Vienna, Austria) and SPSS software v25.0 (SPSS Inc., Chicago, IL).

## Results

### Patients

**Fig. 1:**
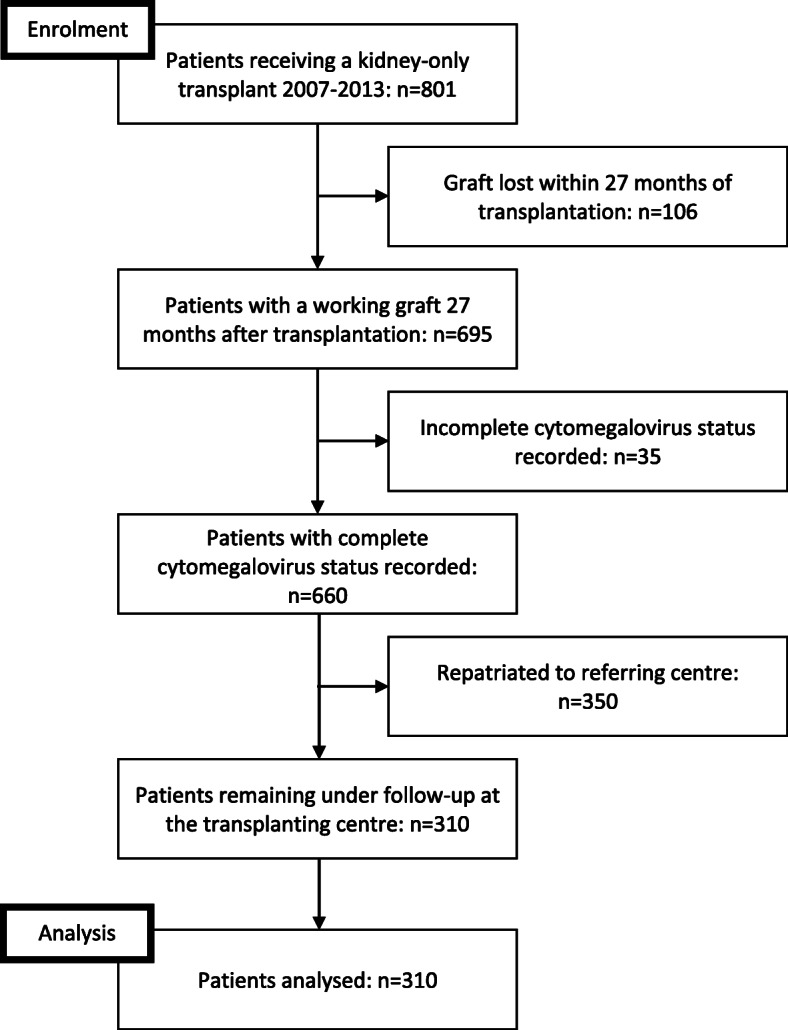
Flow diagram showing numbers and reasons for patients included and excluded in this study

Eight hundred and one incident, unselected adult (≥18 years) kidney transplants were performed in our centre over the study period. Of these, 106 (13%) lost their graft within 27 months of transplantation. Three hundred and fifty patients were repatriated to their referring unit and therefore excluded. A further 35 subjects were excluded for having incomplete CMV serostatus recorded leaving 310 patients in the analyses (Fig. 1). Overall median time post-transplant was 8.0 years (Interquartile range [IQR] 6.6–9.7 years) giving an overall median follow-up period of 5.7 years (IQR 4.4–7.5 years) after the initial 27 months of data collection were excluded. The median number of eGFR measurements available for analysis from 3 months to 27 months after transplantation was 54 (IQR 36–82) with the minimum being 16 measurements.

### Probability of having an episode of rapid deterioration in months 3–27 after kidney transplantation

**Fig. 2:**
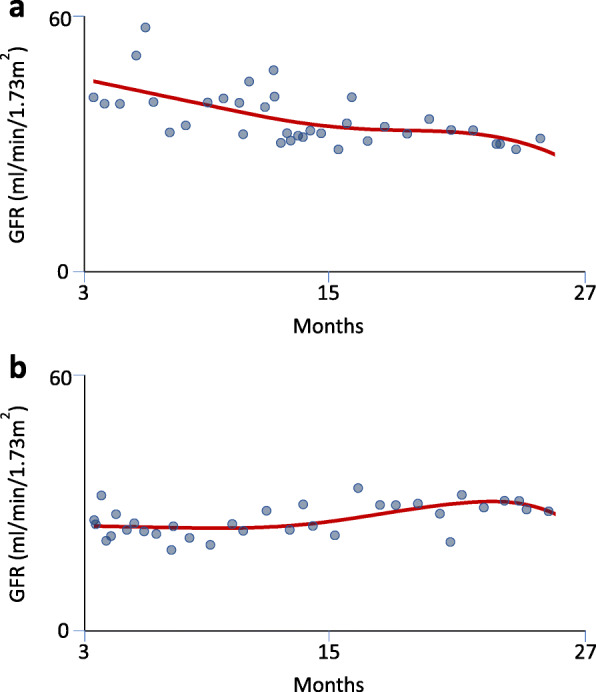
**a** shows an average trajectory with probability close to 1 of rapid deterioration. **b** shows an average trajectory with probability close to 0 of rapid deterioration. The horizontal axis is months since transplantation and the vertical axis is estimated glomerular filtration rate (eGFR; ml/min/1.73 m^2^). (blue dots) eGFR data. (red smooth curve) The estimated trajectory

**Fig. 3:**
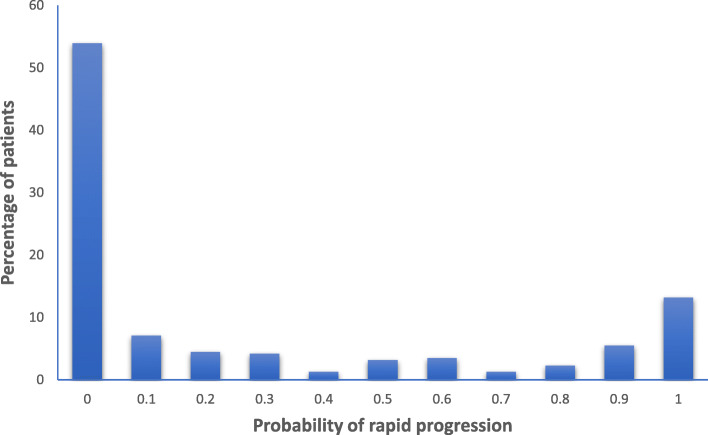
The distribution of probabilities of rapid deterioration visualised by percentage frequency histograms

Figure 2a and b show example trajectory plots for individual patients with probabilities of having an episode of rapid deterioration of 1.0 and 0 respectively. The distribution of probability of a period of rapid deterioration is shown in Fig. 3. Sixty-five patients (21%) had a probability of rapid deterioration ≥0.8 and 90 (29%) had a probability ≥0.5. The clinical demographics of the patients classified as having, or not having ≥0.8 probability of a period of rapid deterioration of renal function are presented in Table 1. Other than CMV serostatus groups, there were only minimal differences between the two groups, and none were statistically significant. Whereas all four CMV serostatus groups were roughly equally represented in patients without rapid deterioration, significantly more patients with a probability of rapid deterioration ≥0.8 were D+/R+ (46%) than D−/R− (12%; *P* = 0.01). Patients with a probability of rapid deterioration ≥0.8 were more likely to be CMV seropositive (CMV+) at the time of transplantation (66.2% vs 48.6%; *P* = 0.012) and more likely to receive a kidney from a CMV+ donor (67.7% vs 50.6%, *P* = 0.017) than patients without rapid deterioration of renal function. Using different cut-offs of probability of rapid deterioration ≥0.7 and 0.9 did not materially affect the results (Supplementary Table 1a and b).

**Table 1:** Comparison of baseline characteristics between groups on estimated glomerular filtration rate trajectories. The presence of an episode of rapid deterioration is based on more than 80% of curves displaying this characteristic. The number (percentage) or mean (standard deviation) or median (25th, 75th quartiles) are reported for each subgroup. The median (range) is reported for the number of eGFR measurements

	Rapid Deterioration		***P***-value
	No (***n*** = 245)	Yes (***n*** = 65)	
Age (years)	45.73 ± 12.96	46.65 ± 14.36	0.622
Male	110 (45%)	25 (39%)	0.400
Race,			
White	168 (68.6%)	49 (75.4%)	0.778
Asian	50 (20.4%)	9 (13.8%)	
Black	15 (6.1%)	4 (6.2%)	
Other	12 (4.9%)	–	
BMI (kg/m^2^)	27.14 (23.44, 30.94)	27.63 (24.00, 31.74)	0.379
Diabetes mellitus	22 (9%)	9 (14%)	0.249
Time on transplant waiting (days)	410 (213–1395)	656 (257–1407)	0.187
IMD			
1	92 (37.6%)	21 (32.8%)	0.825
2	44 (18.0%)	11 (17.2%)	
3	53 (21.6%	18 (28.1%)	
4	24 (9.8%)	7 (10.9%)	
5	32 (13.1%)	7 (10.9%)	
Mean Number of HLA-mismatches	2.80 ± 1.53	2.67 ± 1.37	0.553
NODAT	15 (6.2%)	5 (7.7%)	0.777
Transplant Number			
First	216 (88.2%)	59 (90.8%)	0.720
Second	21 (8.6%)	6 (9.2%)	
Third	5 (2.0%)	0 (0%)	
Donor Type			
Cadaveric	119 (49%)	38 (58%)	0.165
Live	126 (51%)	27 (42%)	
Donor age (years)	44.99 ± 13.29	48.08 ± 13.97	0.110
Male Donor	104 (52%)	31 (54%)	0.765
Donor Race			
White	202 (82.4%)	60 (92.3%)	0.252
Asian	13 (5.3%)	1 (1.5%)	
Black	26 (10.6%)	3 (4.6%)	
Other	4 (1.6%)	1 (1.5%)	
Donor BMI (kg/m^2^)	25.82 (23.02, 28.50)	25.35 (23.29, 28.56)	0.863
CMV Serostatus Combinations			
D−/R−	69 (28%)	8 (12%)	0.010
D−/R+	57 (23%)	14 (22%)	
D+/R−	52 (21%)	13 (20%)	
D+/R+	67 (27%)	30 (46%)	
CMV D+	124 (50.6)	44 (67.7%)	0.017
CMV R+	119 (48.6%)	43 (66.2%)	0.012
Acute rejection in first year	30 (12.4%)	12 (18.5%)	0.206
Delayed graft function	109 (44.5%)	22 (38.5%)	0.402
Number of eGFR measurements	54 (35.0, 83.5)	54 (39.0, 79.5)	0.929
eGFR (ml/min/1.73 m^2^)			
3 months	54.63 ± 19.11	54.31 ± 22.27	0.905
12 months	53.07 ± 20.49	51.92 ± 17.28	0.683
27 months	51.67 ± 19.52	46.71 ± 15.98	0.091

*BMI* Body mass index, *CMV* Cytomegalovirus, *D−* donor CMV seronegative, *D+* Donor CMV seropositive, *eGFR* Estimated glomerular filtration rate, *IMD* Index of multiple deprivation, *NODAT* New onset diabetes after transplantation, *R*-− Recipient CMV seronegative, *R+* Recipient CMV seropositive

### Factors associated with having an episode of rapid deterioration

Univariable and adjusted associations with having a probability of rapid deterioration ≥0.8 are shown in Supplementary Table 2. In ***separate*** multivariable analyses, only CMV D+/R+ (Odds ratio [OR] 3.86; 95% confidence intervals [CI] 1.65–9.03; *P* = 0.002: ref. D−/R−), Donor CMV+ (OR 2.05; 95%CI 1.15–3.64; *P* = 0.015: ref. Donor CMV seronegative [CMV−]) and Recipient CMV+ (OR 2.07; 95%CI 1.17–3.66; *P* = 0.013: ref. Recipient CMV-) were significantly associated with a probability ≥0.8 of having an episode of rapid deterioration of renal function, and were independent of renal function when individually adjusted for eGFR at 27 months. Results were materially unchanged if different cut-offs of probability of rapid deterioration ≥0.7 and 0.9 were used (Supplementary Table 3).

### Death-censored graft loss

**Fig. 4:**
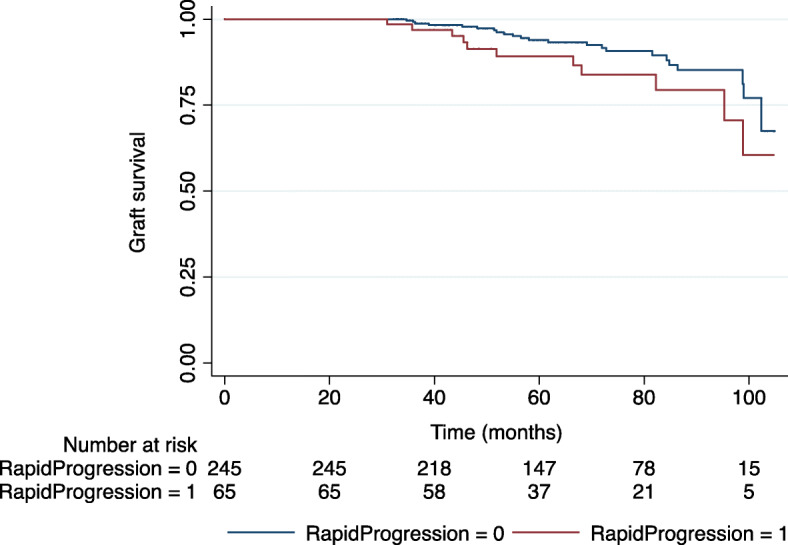
Death-censored Kaplan-Meier graft survival analysis for patients with and without a ≥ 0.8 probability of an episode of rapid deterioration

A total of 34 (10.9%) of patients lost their graft and either started dialysis or were re-transplanted during the follow-up period. The death-censored graft survival curve for patients with and without a ≥ 0.8 probability of an episode of rapid deterioration of renal function is shown in Fig. 4. Although graft loss appeared to be higher in patients (16.9% v. 9.4%) with an episode of rapid deterioration, this did not achieve statistical significance (log-rank test *P* = 0.071) in univariable analysis. The full univariable associations with death-censored graft loss are shown in Supplementary Table 4.

In multivariable Cox regression modelling, having a probability ≥0.8 of an episode of rapid deterioration of renal function (Hazard ratio [HR] 2.38; 95%CI 1.14–4.98; *P* = 0.022), eGFR at 27 months (HR 0.95; 95%CI 0.93–0.98; *P* = 0.002) and recipient age (HR 0.94; 95%CI 0.91–0.97; *P* < 0.001) were significantly associated with death-censored graft-loss. Inclusion of these factors resulted in good model fit versus null model (model coefficient: Chi-square 45.544, *p* < 0.001 for rapid deterioration ≥0.8). Model fit was not materially different if eGFR at 12 months was inserted into the model or if different cut-offs of probability of rapid deterioration ≥0.7 and 0.9 were used (model coefficient: Chi-square 48.100, *p* < 0.001 and Chi-square 47.207, *p* < 0.001 versus null model for rapid deterioration ≥0.7 and 0.9, respectively) (Supplementary Table 5a and b).

### All-cause mortality

A total of 43 (13.9%) of patients died during the follow-up period. Of these 10 had a probability of an episode of rapid deterioration of renal function greater than 0.8 and 30 did not (*P* = 0.689). The full univariable associations with all-cause mortality are shown in Supplementary Table 6. Having a probability of rapid deterioration (HR 1.22; 95%CI 0.60–2.48; *P* = 0.578) greater than 0.8 was not associated with all-cause mortality. In multiple regression models, only recipient age (HR 1.07; 95%CI 1.03–1.11; *P* = 0.001) and eGFR at the beginning of the follow-up period (HR 0.97; 95%CI 0.95–1.00: *P* = 0.028) remained significantly associated with all-cause mortality. Results were materially unchanged using cut-offs of probability ≥0.7 or 0.9 for rapid deterioration (Supplementary Table 7).

## Discussion

We have previously shown in a prevalent renal transplant recipient cohort that an episode of rapid deterioration of renal function is associated with subsequent graft loss [16]. In this study we extend these findings, using the same Bayesian smoothing technique, to show that in an unselected, incident renal transplant cohort, an early episode of rapid deterioration is associated with death-censored graft loss in multivariable regression models. Furthermore, our findings demonstrate that CMV seropositivity is associated with episodes of rapid deterioration of renal function raising the possibility of a mechanism that could theoretically provide a therapeutic target to extend graft survival.

Whether the donor or recipient were CMV seropositive, and especially if both were seropositive, was associated with an increased probability of having at least one episode of rapid deterioration of renal function loss between 3 and 27 months after transplantation. Having a high probability of an episode of rapid deterioration was in turn, significantly associated with death-censored graft loss. These associations persisted after adjusting for eGFR at 27 months, suggesting the relationship between episodes of rapid deterioration early in the post-transplant period and graft loss is not mediated by having a lower eGFR at the start of the observation/follow-up period. These findings also suggest that CMV seropositivity may explain the relationship between early episodes of rapid deterioration of renal function and graft loss, although the observational nature of our study cannot confirm this.

Infection is a major cause of morbidity and mortality in solid organ transplantation [18]. The risk is further increased in the presence of CMV seropositivity [19], likely due to interaction between the virus and host immune response [20]. In anti-neutrophil cytoplasmic antibody-associated vasculitis, a condition associated with increased infection-related mortality, the presence of subclinical CMV infection appeared to adversely affect the available functional CD4^+^ T-cell compartment, resulting in impaired immunity to other antigens [21]. Therefore, a potential explanation for the association between CMV seropositivity and episodes of rapid deterioration may be mediated by more frequent intercurrent infections. This hypothesis cannot be addressed by this current study but warrants further investigation.

An alternative explanation is that clinically symptomatic CMV infection itself, either recurrent or primary, is associated with episodes of rapid deterioration of renal function. However, rates of post-transplant symptomatic CMV infection are generally low [22, 23], and have reduced further by 50–70% with intravenous/oral ganciclovir prophylaxis in trials of CMV D+ renal transplants [24, 25]. Perhaps more common than symptomatic infection are recurrent, clinically undetected episodes of CMV reactivation. In paediatric and young adult renal transplant cohorts, subclinical CMV infection occurred in 22% [26], and resulted in greater odds of acute rejection, chronic allograft injury and up to 30% lower eGFR [26, 27]. Shabir et al. [28] demonstrated that CD4^+^CD28^null^ T-cells were found predominantly in CMV R+ kidney transplants, which, in turn, were associated with delayed graft function and poorer allograft function at 12 months, and in vitro glomerular endothelial cell injury. This, again, highlights the detrimental immunomodulatory effects of the CMV virus. The rates of subclinical and symptomatic CMV infection were unavailable at the time of this analysis and would be an intriguing line of investigation for a future study using the Bayesian technique.

In our cohort, an episode of acute rejection in the first year was not associated with having an episode of rapid decline. There were fewer patients who had acute rejection in the group who had an episode of rapid decline compared to the group who did not, although this was not statistically significant. A possible explanation may be that the Bayesian methodology smoothed out sharp declines followed by rapid, treatment-related improvement to produce averaged trajectories which did not meet the predefined criteria for an episode of rapid deterioration. An advantage of this Bayesian technique is that it smooths out short-term variations in eGFR that might arise from concurrent infectious episodes or dehydration episodes, or indeed any events that could cause short-term reductions in renal function. It, therefore, remains a possibility for CMV seropositivity to be a contributing factor leading to rapid deterioration of renal function through an episode of rejection. Several studies have demonstrated this association [29–32]. Whilst not all studies recapitulated these findings [33], several potential CMV-associated mechanisms have been described, including but not limited to increased expression of major histocompatibility class I and II molecules on vascular and tubular cells through production of T-cell derived interferon-γ [34, 35], elevated anti-endothelial cell antibodies and interleukin-2 levels [36], and enhanced production of co-stimulatory molecules on vascular endothelial, tubular epithelial and T-cells [37, 38].

Another potential mechanism linking CMV to graft dysfunction can be found in cardiac transplantation. Cardiac graft vasculopathy is a major determinant of long-term graft survival [39] and CMV infection an established risk factor [38, 40–42]. Other studies have shown an association between CMV and systemic arteriosclerosis [43, 44], and atherosclerotic events in renal transplant recipients [45]. Whether CMV is a significant risk factor for renal allograft vasculopathy requires further examination.

In our study, D+/R+ transplants had a higher odds ratio than D+/R− transplants of having a probability of rapid deterioration ≥0.8, suggesting that D+/R− transplants would have a better graft survival outcome. This is in contrast to published evidence by Leeaphorn et al., reporting worse graft survival in D+/R− compared to other serotype pairings [7]. This discrepancy may be attributable to the CMV prophylaxis strategy used at our centre during the time of transplantation (2007–2013): only CMV D+/R− patients had valganciclovir prophylaxis. CMV D+/R+ patients were not treated exposing them to the risk of CMV viraemia.

Taken together, our findings and those of Leeaphorn et al. suggest that extending CMV prophylaxis to D−/R+ and D+/R+ pairings could potentially increase graft survival. We acknowledge, however, that our study does not provide direct evidence to support this and further evidence is required before such a change in practice is considered. Since 2014, all CMV D+ transplants in our centre have received valganciclovir prophylaxis in accordance with consensus guidelines [46]. Repeating our study in a post-2014 renal transplant incident cohort would reveal whether CMV prophylaxis improves outcomes for patients receiving CMV seropositive kidneys.

Our study has some limitations. The analysis was retrospective, and the available dataset was not able to provide granular information regarding potential causes of rapid deterioration of renal function (e.g. renal pathology reports, CMV viral loads, changes in immunosuppression). Furthermore, because of the granularity of data required in the early observation period, patients who were repatriated to their referring centre after their successful kidney transplant could not be included in this study. This could have led to some further confounding of our study findings and needs to be explored in a larger subsequent study. Unfortunately, hospitals in the United Kingdom and indeed many other countries, have their own EHR with different levels of accessibility and granularity of data. As such, future larger studies will need to allow enough resource and time to allow data to be collected from different systems and be integrated for analysis. Until then, studies of the type presented here will be relatively small and limited to a single centre. Finally, consideration may be given for the inclusion of composite co-morbidity scores such as the Charlson comorbidity index in future studies to reduce other potential confounders not captured by the current data in all-cause mortality analysis.

However, our study also has several strengths. Leveraging the power of EHR in our centre, we had numerous eGFR measurements per patient and an extended follow-up period. The use of modern Bayesian modelling has been combined with clinically relevant and recognised definition of rapid deterioration to provide a rigorous assessment of renal function trajectory. It should be recognised that there are other methods for analysing large amounts of continuous data being developed, including, but not limited to, joint mixed models [47]. Each of these methods have their own strengths and weaknesses. Their use will very much depend on the questions being considered.

## Conclusion

Our study further validates the utility of using novel statistical techniques to robustly model renal allograft function to improve prediction of renal transplant trajectory, and to better understand the factors influencing these trajectories. Additional efforts and larger prospective studies examining renal allograft function trajectories will be required to identify whether current efforts to manage subclinical and occult CMV are sufficient in improving long-term graft survival. The aim is to better manage patients with specific trajectories and risk factors, and to allow more timely interventions and counselling, especially if re-transplantation is a possibility.

## Supplementary Information

**Additional file 1.** Figures 1, 2a, 2b, 3 and 4.

**Additional file 2.** Additional file containing Supplementary Tables as referenced in-text.

## Data Availability

The data that support the findings of this study are available from the corresponding author, CJF, upon reasonable request.

## Abbreviations

BMI: Body mass index
CI: Confidence interval
CKD: Chronic kidney disease
CMV: Cytomegalovirus
CMV+: Cytomegalovirus seropositive.
CMV−: Cytomegalovirus seronegative
D+: Donor cytomegalovirus seropositive.
D−: Donor cytomegalovirus seronegative
eGFR: Estimated glomerular filtration rate
EHR: Electronic health record
ESRD: End stage renal disease
HR: Hazard ratio
IMD: Index of multiple deprivation
IQR: Interquartile range
NODAT: New onset diabetes after transplantation
OR: Odds ratio
R+: Recipient cytomegalovirus seropositive.
R−: Recipient cytomegalovirus seronegative
