# Supplementary figures and images for: Early renal function trajectories, cytomegalovirus serostatus and long-term graft outcomes in kidney transplant recipients
Source: BMC Nephrol. 2021 Mar 20;22:102. doi: 10.1186/s12882-021-02285-2 (PMC7981965; doi:10.1186/s12882-021-02285-2)

**Figure 1.**

**Figure 2a.**


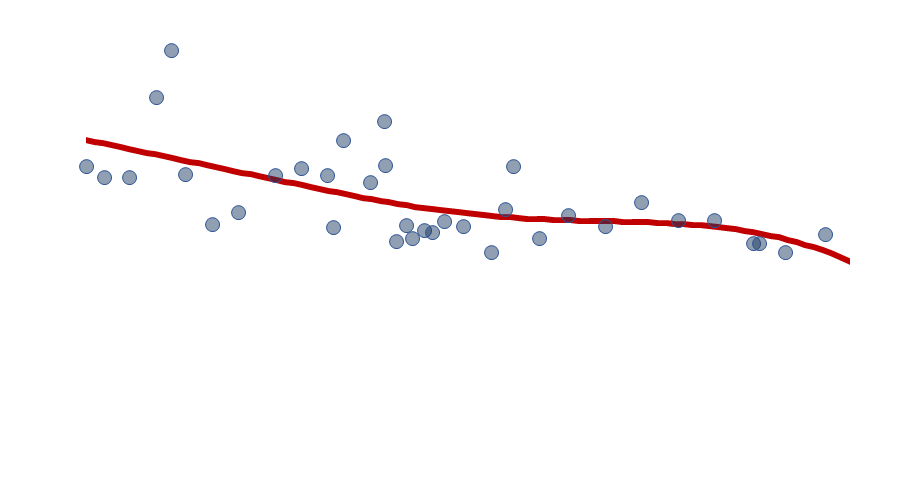


3

15

0

60

GFR (ml/min/1.73m2)

27

Months

**Figure 2b.**


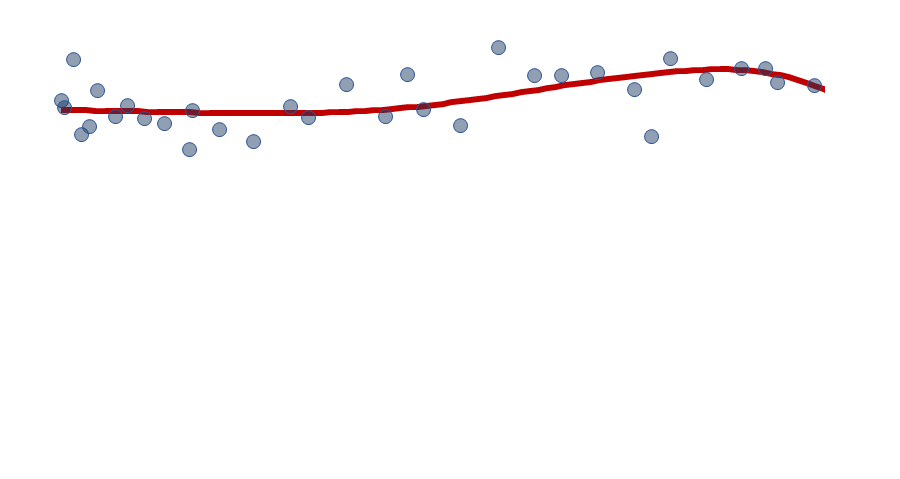


3

15

Months

0

60

GFR (ml/min/1.73m2)

27

**Figure 3.**

**Figure 4.**

Supplement: Supplementary file 1 — Additional file 1. Figures 1, 2a, 2b, 3 and 4. [file 12882_2021_2285_MOESM1_ESM.docx]
