# Supplementary material for: Early renal function trajectories, cytomegalovirus serostatus and long-term graft outcomes in kidney transplant recipients
Source: BMC Nephrol. 2021 Mar 20;22:102. doi: 10.1186/s12882-021-02285-2 (PMC7981965; doi:10.1186/s12882-021-02285-2)
Supplement: Supplementary file 2 — Additional file 2. Additional file containing Supplementary Tables as referenced in-text. [file 12882_2021_2285_MOESM2_ESM.docx]

**Supplementary Table 1a.** Comparison of baseline characteristics between groups on estimated glomerular filtration rate trajectories. The presence of an episode of rapid deterioration is based on more than 70% of curves displaying this characteristic.

|  | **Rapid Deterioration** | |  |
| --- | --- | --- | --- |
|  | **No**  **(n=241)** | **Yes**  **(n=69)** | **P-value** |
| Age (years) | 45.77 ± 12.96 | 46.48 ± 14.249 | 0.695 |
| Male | 109 (45.2%) | 26 (37.7%) | 0.275 |
| Race,  White  Asian  Black  Other | 172 (68.3%)  51 (20.2%)  15 (6%)  14 (5.6%) | 45 (77.6%)  8 (13.8%)  4 (6.9%)  1 (1.7%) | 0.558 |
| BMI (kg/m^2^) | 27.26 (23.44, 30.94) | 27.60 (24.00, 31.74) | 0.418 |
| Diabetes mellitus | 21 (8.7%) | 10 (14.5%) | 0.174 |
| Time on transplant waiting (days) | 473 (220, 1414) | 638 (256, 1380) | 0.388 |
| IMD  1  2  3  4  5 | 91 (37.8%)  43 (17.8%)  51 (21.2%)  24 (10.0%)  32 (13.3%) | 22 (32.4%)  12 (17.6%)  20 (29.4%)  7 (10.3%)  7 (10.3%) | 0.683 |
| Mean Number of HLA-mismatches | 2.80 ± 1.52 | 2.67 ± 1.42 | 0.561 |
| NODAT | 14 (5.9%) | 6 (8.7%) | 0.410 |
| Transplant Number  First  Second  Third | 213 (89.5%)  20 (8.4%)  5 (2.1%) | 62 (89.9%)  7 (10.1%)  0 (0%) | 0.583 |
| Donor Type  Cadaveric  Live | 119 (49.4%)  122 (50.6%) | 38 (55.1%)  31 (44.9%) | 0.416 |
| Donor age (years) | 45.02 ± 13.29 | 47.77 ± 13.98 | 0.146 |
| Male Donor | 102 (51.8%) | 33 (54.1%) | 0.771 |
| Donor Race  White  Asian  Black  Other | 199 (82.6%)  25 (10.4%)  13 (5.4%)  4 (1.7%) | 63 (91.4%)  4 (5.8%)  1 (1.4%)  1(1.4%) | 0.327 |
| Donor BMI (kg/m^2^) | 25.35 (23.29, 28.72) | 25.82 (23.02, 28.42) | 0.998 |
| CMV Serostatus Combinations  D-/R-  D-/R+  D+/R-  D+/R+ | 66 (27.4%)  57 (23.7%)  52 (21.6%)  66 (27.4%) | 11 (15.9%)  14 (20.3%)  13 (18.8%)  31 (44.9%) | 0.041 |
| CMV D+ | 123 (51.0%) | 45 (65.2%) | 0.040 |
| CMV R+ | 118 (49.0%) | 44 (63.8%) | 0.040 |
| Acute rejection in first year | 28 (11.8%) | 14 (20.3%) | 0.076 |
| Delayed graft function | 105 (43.6%) | 29 (42.0%) | 0.891 |
| Number of eGFR measurements | 56.0 (40.0, 80.5) | 53 (35.0, 82,5) | 0.532 |
| eGFR (ml/min/1.73m^2^)  3 months  12 months  27 months | 54.70 ± 19.20  53.36 ±20.52  51.40 ± 19.56 | 54.10 ± 19.10  50.97 ± 17.27  46.16 ± 15.85 | 0.819  0.390  0.042 |

The number (percentage) or mean (standard deviation) or median (25^th^, 75^th^ quartiles) are reported for each subgroup. The median (range) is reported for the number of eGFR measurements.

BMI, body mass index; CMV, cytomegalovirus; D-, donor CMV seronegative, D+, donor CMV seropositive; eGFR, estimated glomerular filtration rate; IMD, index of multiple deprivation; NODAT, new onset diabetes after transplantation; R-, recipient CMV seronegative; R+, recipient CMV seropositive.

**Supplementary Table 1b.** Comparison of baseline characteristics between groups on estimated glomerular filtration rate trajectories. The presence of an episode of rapid deterioration is based on more than 90% of curves displaying this characteristic.

|  | **Rapid Deterioration** | |  |
| --- | --- | --- | --- |
|  | **No**  **(n=252)** | **Yes**  **(n=58)** | **P-value** |
| Age (years) | 45.64 ± 13.09 | 37.17 ± 13.92 | 0.427 |
| Male | 252 (45.6%) | 58 (34.5%) | 0.143 |
| Race,  White  Asian  Black  Other | 164 (68%)  50 (20.7%)  15 (6.2%)  12 (5.0%) | 53 (76.8%)  9 (13.0%)  (5.8%)  3 (4.3%) | 0.627 |
| BMI (kg/m^2^) | 27.89 (24.23, 31.64) | 26.95 (23.42, 30.94) | 0.370 |
| Diabetes mellitus | 22 (8.7%) | 9 (15.5%) | 0.144 |
| Time on transplant waiting (days) | 429 (221, 1412) | 636 (225, 1387) | 0.283 |
| IMD  1  2  3  4  5 | 94 (37.3%)  45 (16.9%)  54 (21.4%)  27 (10.7%)  32 (12.7%) | 19 (33.3%)  10 (17.5%)  17 (29.8%)  4 (7.0%)  7 (12.3%) | 0.720 |
| Mean Number of HLA-mismatches | 2.76 ± 1.54 | 2.81 ± 1.31 | 0.845 |
| NODAT | 15 (6.0%) | 5 (8.6%) | 0.553 |
| Transplant Number  First  Second  Third | 223 (89.6%)  21 (8.4%)  5 (2.0%) | 52 (89.7%)  6 (10.3%)  0 (0%) | 0.635 |
| Donor Type  Cadaveric  Live | 124 (49.2%)  128 (50.8%) | 33 (56.9%)  25 (43.1%) | 0.311 |
| Donor age (years) | 44.75 ± 13.46 | 49.45 ± 12.99 | 0.020 |
| Male Donor | 107 (51.4%) | 28 (56.0%) | 0.637 |
| Donor Race  White  Asian  Black  Other | 209 (82.9%)  13 (5.2%)  26 (10.3%)  4 (1.6%) | 53 (91.4%)  3 (5.2%)  1 (1.7%)  1 (1.7%) | 0.421 |
| Donor BMI (kg/m^2^) | 25.15 (23.02, 28.82) | 25.82 (23.10, 28.43) | 0.761 |
| CMV Serostatus Combinations  D-/R-  D-/R+  D+/R-  D+/R+ | 70 (27.8%)  60 (23.8%)  55 (21.8%)  67 (26.6%) | 7 (12.1%)  11 (19.0%)  65 (21.0%)  97 (31.3%) | 0.002 |
| CMV D+ | 127 (50.4%) | 41 (70.7%) | 0.005 |
| CMV R+ | 122 (48.84%) | 40 (69.0) | 0.005 |
| Acute rejection in first year | 30 (12.0%) | 12 (20.7%) | 0.092 |
| Delayed graft function | 112 (44.4%) | 22 (37.9%) | 0.382 |
| Number of eGFR measurements | 55.0 (40.0, 79.3) | 53.5 (35.0, 83.7) | 0.852 |
| eGFR (ml/min/1.73m^2^)  3 months  12 months  27 months | 54.70 ± 19.31  53.23 ±20.70  51.08 ± 19.55 | 54.00 ± 18.56  51.04 ± 15.55  46.55 ± 15.37 | 0.805  0.461  0.100 |

The number (percentage) or mean (standard deviation) or median (25^th^, 75^th^ quartiles) are reported for each subgroup. The median (range) is reported for the number of eGFR measurements.

BMI, body mass index; CMV, cytomegalovirus; D-, donor CMV seronegative, D+, donor CMV seropositive; eGFR, estimated glomerular filtration rate; IMD, index of multiple deprivation; NODAT, new onset diabetes after transplantation; R-, recipient CMV seronegative; R+, recipient CMV seropositive.

**Supplementary Table 2.** Univariable and multivariable associations with having a probability of rapid deterioration greater than 0.8.

|  | **Unadjusted OR (95% CI)** | **P-value** | **Adjusted OR (95% CI)** | **P-value** |
| --- | --- | --- | --- | --- |
| Age (years) | 1.01 (0.99-1.03) | 0.621 |  |  |
| Male | 0.77 (0.44-1.34) | 0.353 |  |  |
| Race  White  Asian  Black  Other | 1.0 (reference)  0.62 (0.28-1.34) 0.91 (0.29-2.88)  0.94 (0.25-3.49) | -  0.224  0.878  0.920 |  |  |
| BMI (kg/m^2^) | 1.03 (0.98-1.08) | 0.274 |  |  |
| Diabetes mellitus | 1.63 (0.71-3.73) | 0.249 |  |  |
| Time on transplant waiting (days) | 1.00 (1.00-1.00) | 0.233 |  |  |
| IMD  1  2  3  4  5 | 1.0 (reference)  1.10 (0.49-2.45)  1.49 (0.73-3.04)  1.28 (0.49-3.36)  0.96 (0.37-2.47) | -  0.826  0.276  0.619  0.930 |  |  |
| Mean Number of HLA-mismatches | 0.94 (0.76-1.12) | 0.574 |  |  |
| NODAT | 0.79 (0.28-2.27) | 0.665 |  |  |
| Previous transplant | 0.75 (0.33-1.71) | 0.494 |  |  |
| Donor Type  Cadaveric  Live | 1.0 (reference)  0.67 (0.39-1.17) | -  0.158 |  |  |
| Donor Age (years) | 1.02 (1.00-1.04) | 0.111 |  |  |
| Male Donor | 1.11 (0.62-2.01) | 0.724 |  |  |
| Donor Race  White  Asian  Black  Other | 1.0 (reference)  0.51 (0.17-1.51)  0.24 (0.03-1.89)  0.79 (0.09-7.20) | -  0.221  0.177  0.834 |  |  |
| Donor BMI (kg/m^2^) | 0.99 (0.93-1.05) | 0.737 |  |  |
| CMV Serostatus Combinations  D-/R-  D-/R+  D+/R-  D+/R+ | 1. (reference)   2.12 (0.83-5.40)  2.16 (0.83-5.58)  3.86 (1.65-9.03) | **-**  0.116  0.113  0.002 | 1.0 (reference)  2.16 (0.84-5.54)  2.13 (0.82-5.54)  3.82 (1.63-8.97) | -*  0.108  0.120  0.002 |
| *CMV D+* | *2.05 (1.15-3.64)* | *0.015* | *2.06 (1.15-3.68)* | *0.014** |
| *CMV R+* | *2.07 (1.17-3.66)* | *0.013* | *2.03 (1.14-3.60)* | *0.016** |
| Acute rejection in first year | 1.60 (0.77-3.33) | 0.209 |  |  |
| Delayed graft function | 0.78 (0.45-1.37) | 0.384 |  |  |
| eGFR (ml/min/1.73m^2^)  3 months  12 months  27 months | 1.00 (0.99-1.01)  1.00 (0.98-1.01)  0.92 (0.97-1.00) | 0.905  0.684  0.092 | 0.99 (0.97-1.00) | 0.111** |

BMI, body mass index; CMV, cytomegalovirus; CI, confidence intervals; D-, donor CMV seronegative, D+, donor CMV seropositive; eGFR, estimated glomerular filtration rate; IMD, index of multiple deprivation; NODAT, new onset diabetes after transplantation; R-, recipient CMV seronegative; R+, recipient CMV seropositive; OR, odds ratio.

* Adjusted for eGFR at 27 months. CMV serostatus combinations, CMV D+ and CMV R+ were entered individually in ***separate*** multivariable models.

**Adjusted for CMV serostatus combinations and acute rejection at 1 year

**Supplementary Table 3.** Univariable and multivariable associations with having a probability of rapid deterioration greater than 0.7 and 0.9.

|  | **Rapid Deterioration** ≥**0.7** | |  |  |
| --- | --- | --- | --- | --- |
|  | **Unadjusted OR (95% CI)** | **P-value** | **Adjusted OR (95% CI)** | **P-value** |
| Age | 1.00 (0.98-1.03) | 0.694 |  |  |
| Male | 0.73 (0.42-1.27) | 0.266 |  |  |
| Race  White  Asian  Black  Other | 1.0 (reference)  0.83 (0.26-2.60)  0.58 (0.26-1.21)  0.84 (0.23-3.14) | -  0.742  0.139  0.800 |  |  |
| BMI (kg/m^2^) | 1.03 (0.98-1.08) | 0.312 |  |  |
| Diabetes mellitus | 1.78 (0.79-3.98) | 0.163 |  |  |
| Time on transplant waiting (days) | 1.00 (1.00-1.00) | 0.498 |  |  |
| IMD  1  2  3  4  5 | 1.0 (reference)  1.15 (0.52-2.55)  1.62 (0.81-3.25)  1.21 (0.46-3.16)  0.91 (0.35-2.32) | -  0.722  0.173  0.702  0.835 |  |  |
| Mean Number of HLA-mismatches | 0.94 (0.76-1.16) | 0.559 |  |  |
| NODAT | 1.52 (0.56-4.13) | 0.407 |  |  |
| Previous transplant | 0.96 (0.40-2.33) | 0.931 |  |  |
| Donor Type  Cadaveric  Live | 1.0 (reference)  0.80 (0.47-1.36) | -  0.405 |  |  |
| Donor Age (years) | 1.02 (1.00-1.04) | 0.147 |  |  |
| Male Donor | 1.10 (0.62-1.95) | 0.751 |  |  |
| Donor Race  White  Asian  Black  Other | 1.0 (reference)  0.51 (0.17-1.51)  0.24 (0.03-1.89)  0.79 (0.09-7.20) | -  0.221  0.177  0.834 |  |  |
| Donor BMI (kg/m^2^) | 0.99 (0.94-1.05) | 0.801 |  |  |
| CMV Serostatus Combinations  D-/R-  D-/R+  D+/R-  D+/R+ | 1.0 (reference)  1.47 (0.62-3.50)  1.50 (0.62-3.62)  2.82 (1.31-6.07) | -  0.380  0.367  0.008 | 1.0 (reference)  1.45 (0.60-3.46)  1.41 (0.58-3.43)  2.53 (1.16-5.52) | -^1^  0.409  0.448  0.020 |
| *CMV D+* | *1.80 (1.03-3.14)* | *0.039* | *1.68 (0.96-2.95)* | *0.069^1^* |
| *CMV R+* | *1.83 (1.06-3.19)* | *0.031* | *1.72 (0.98-3.01)* | *0.061^1^* |
| Acute Rejection at 1 year | 1.91 (0.94-3.87) | 0.073 | 1.49 (0.71-3.12) | 0.289^2^ |
| Delayed graft function | 0.94 (0.55-1.61) | 0.820 |  |  |
| eGFR (ml/min/1.73m^2^)  3 months  12 months  27 months | 1.00 (0.98-1.01)  0.99 (0.98-1.01)  0.99 (0.97-1.00) | 0.818  0.994  0.043 | 0.99 (0.97-1.00) | 0.074^3^ |

|  | **Rapid Deterioration** ≥**0.9** | |  |  |
| --- | --- | --- | --- | --- |
|  | **Unadjusted OR (95% CI)** | **P-value** | **Adjusted OR (95% CI)** | **P-value** |
| Age | 1.01 (0.99-1.03) | 0.426 |  |  |
| Male | 0.63 (0.35-1.14) | 0.124 |  |  |
| Race  White  Asian  Black  Other | 1.0 (reference)  1.02 (0.32-3.22)  0.60 (0.27-1.35)  0.29 (0.04-2.31) | -  0.974  0.218  0.244 |  |  |
| BMI (kg/m^2^) | 1.02 (0.97-1.07) | 0.506 |  |  |
| Diabetes mellitus | 1.92 (0.83-4.42) | 0.125 |  |  |
| Time on transplant waiting (days) | 1.00 (1.00-1.00) | 0.314 |  |  |
| IMD  1  2  3  4  5 | 1.0 (reference)  1.10 (0.47-2.56)  1.56 (0.75-3.25)  0.73 (0.23-2.34)  1.08 (0.42-2.81) | -  0.826  0.237  0.600  0.871 |  |  |
| Mean Number of HLA-mismatches | 1.02 (0.82-1.28) | 0.844 |  |  |
| NODAT | 1.47 (0.51-4.23) | 0.473 |  |  |
| Previous transplant | 0.99 (0.39-2.53) | 0.983 |  |  |
| Donor Type  Cadaveric  Live | 1.0 (reference)  0.73 (0.41-1.31) | -  0.292 |  |  |
| Donor Age (years) | 1.03 (1.00-1.05) | 0.021 | 1.03 (1.00-1.05) | 0.036^4^ |
| Male Donor | 1.20 (0.65-2.24) | 0.563 |  |  |
| Donor Race  White  Asian  Black  Other | 1.0 (reference)  0.46 (0.13-1.56)  0.30 (0.04-2.37)  0.99 (0.11-9.00) | -  0.210  0.255  0.990 |  |  |
| Donor BMI (kg/m^2^) | 0.99 (0.94-1.06) | 0.852 |  |  |
| CMV Serostatus Combinations  D-/R-  D-/R+  D+/R-  D+/R+ | 1.0 (reference)  1.83 (0.70-5.03)  1.82 (0.65-5.01)  4.48 (1.84-10.89) | -  0.239  0.255  0.001 | 1.0 (reference)  1.53 (0.55-4.32)  1.60 (0.57-4.55)  4.08 (1.63-10.21) | -^5^  0.418  0.375  0.003 |
| CMV D+ | 2.37 (1.29-4.35) | *0.006* | 2.28 (1.20-4.33) | *0.012^5^* |
| CMV R+ | 2.37 (1.28-4.40) | *0.006* | 2.18 (1.15-4.12) | *0.017^5^* |
| Acute Rejection at 1 year | 1.90 (0.91-4.00) | 0.088 | 1.52 (0.66-3.50) | 0.326^6^ |
| Delayed graft function | 0.76 (0.43-1.37) | 0.367 |  |  |
| eGFR (ml/min/1.73m^2^)  3 months  12 months  27 months | 1.00 (0.98-1.01)  0.99 (0.98-1.01)  0.99 (0.97-1.00) | 0.804  0.460  0.101 |  |  |

^1^Adjuusted for acute rejection at 1 year and GFR at 27 months.

^2^Adjusted for CMV serostatus combinations and GFR at 27 months

^3^Adjusted for CMV serostatus combinations and acute rejection at 1 year

^4^Adjusted for CMV serostatus and acute rejection at one year

^5^Adjusted for acute rejection at one year and donor age

^6^Adjusted for CMV serostatus combination and donor age

BMI, body mass index; CMV, cytomegalovirus; CI, confidence intervals; D-, donor CMV seronegative, D+, donor CMV seropositive; eGFR, estimated glomerular filtration rate; IMD, index of multiple deprivation; NODAT, new onset diabetes after transplantation; R-, recipient CMV seronegative; R+, recipient CMV seropositive; OR, odds ratio.

**Supplementary Table 4.** Univariable associations with death-censored graft loss.

|  | **Unadjusted HR (95% CI)** | **P-value** |
| --- | --- | --- |
| Age (years) | 0.98 (0.95-1.00) | 0.074 |
| Male | 1.34 (0.69-2.64) | 0.390 |
| Race  White  Asian  Black  Other | 1.0 (reference)  1.03 (0.42-2.53)  2.40 (0.82-6.96)  2.26 (0.52-9.78) | -  0.954  0.108  0.276 |
| BMI (kg/m^2^) | 1.00 (0.93-1.06) | 0.922 |
| Diabetes mellitus | 1.99 (0.83-4.81) | 0.126 |
| Time on transplant waiting (days) | 1.00 (1.00-1.00) | 0.197 |
| IMD  1  2  3  4  5 | 1.0 (reference)  1.02 (0.41-2.53)  0.80 (0.32-1.98)  0.74 (0.21-2.58)  0.57 (0.16-1.99) | -  0.963  0.627  0.740  0.572 |
| Mean Number of HLA-mismatches | 1.05 (0.79-1.39) | 0.743 |
| NODAT | 2.55 (0.99-6.59) | 0.053 |
| Previous transplant | 1.11 (0.49-2.49) | 0.805 |
| Donor Type  Cadaveric  Live | 1.0 (reference)  0.82 (0.41-1.6) | -  0.553 |
| Donor Age (years) | 1.01 (0.99-1.04) | 0.332 |
| Male Donor | 1.54 (0.73-3.25) | 0.262 |
| Donor Race  White  Asian  Black  Other | 1.0 (reference)  1.04 (0.13-8.46)  2.37 (1.03-5.45)  2.28 (0.31-16.85) | -  0.973  0.043  0.419 |
| Donor BMI (kg/m^2^) | 1.00 (0.93-1.08) | 0.977 |
|  |  |  |
| CMV Serostatus Combinations  D-/R-  D-/R+  D+/R-  D+/R+ | 1.0 (reference)  0.53 (0.16-1.76)  1.42 (0.55-3.67)  1.33 (0.55-3.20) | -  0.301  0.473  0.530 |
| CMV D+ | 0.83 (0.42-1.62) | 0.582 |
| CMV R+ | 1.76 (0.87-3.56) | 0.114 |
| Acute rejection in first year | 1.38 (0.57-3.33) | 0.478 |
| Delayed graft function | 1.71 (0.87-3.34) | 0.120 |
| eGFR (ml/min/1.73m^2^)  3 months  12 months  27 months | 0.99 (0.97-1.01)  0.96(0.93-0.98)  0.95 (0.93-0.97) | 0.303  <0.001  <0.001 |
| RD >0.8 | 1.92 (0.93-3.93) | 0.077 |

BMI, body mass index; CMV, cytomegalovirus; CI, confidence intervals; D-, donor CMV seronegative, D+, donor CMV seropositive; eGFR, estimated glomerular filtration rate; IMD, index of multiple deprivation; NODAT, new onset diabetes after transplantation; R-, recipient CMV seronegative; R+, recipient CMV seropositive; RD >0.8, greater than 0.8 probability of an episode of rapid deterioration; HR, hazards ratio.

**Supplementary Table 5a.** Multivariate associations with death-censored graft loss using probability of greater than 0.7 of having an episode of rapid deterioration.

Model fit: Chi-squared 48.100, p<0.0001 versus null model.

|  | **Adjusted HR (95% CI)** | **P-value** |
| --- | --- | --- |
| Recipient Age (years) | 0.94 (0.91-0.97) | <0.001 |
| RD >0.7 | 1.97 (0.95-4.07) | 0.069 |
| eGFR (ml/min/1.73m^2^)  12 months  27 months | 0.97 (0.94-1.01)  0.94 (0.92-0.96) | 0.109  <0.001 |
| NODAT | 1.74 (0.65-4.66) | 0.267 |

eGFR, estimated glomerular filtration rate; RD >0.7, greater than 0.7 probability of having an episode of rapid deterioration; HR, hazards ratio; NODAT, new onset diabetes after transplantation.

**Supplementary Table 5b.** Multivariate associations with death-censored graft loss using probability of greater than 0.9 of having an episode of rapid deterioration

Model fit: Chi-squared 47.207, p<0.001 versus null model

|  | **Adjusted HR (95% CI)** | **P-value** |
| --- | --- | --- |
| Recipient Age (years) | 0.94 (0.91-0.97) | <0.001 |
| RD >0.9 | 1.86 (0.85-4.05) | 0.121 |
| eGFR (ml/min/1.73m^2^)  12 months  27 months | 0.98 (0.95-1.01)  0.94 (0.92-0.96) | 0.110  <0.001 |
| NODAT | 1.92 (0.72-5.08) | 0.190 |

eGFR, estimated glomerular filtration rate; RD >0.9, greater than 0.9 probability of having an episode of rapid deterioration; HR, hazards ratio; NODAT, new onset diabetes after transplantation.

**Supplementary Table 6.** Univariable associations with all-cause mortality.

|  | **Unadjusted HR (95% CI)** | **P-value** |
| --- | --- | --- |
| Age (years) | 1.07 (1.04-1.11) | <0.001 |
| Male | 0.998 (0.55-1.82) | 0.996 |
| Race  White  Asian  Black  Other | 1.0 (reference)  0.69 (0.29-1.65)  1.53 (0.54-1.32)  1.00 (1.00-1.00) | -  0.401  0.423  0.976 |
| BMI (kg/m^2^) | 0.98 (0.92-1.04) | 0.496 |
| Diabetes mellitus | 1.54 (0.65-3.66) | 0.326 |
| Time on transplant waiting (days) | 1.00 (1.00-1.00) | 0.004 |
| IMD  1  2  3  4  5 | 1. (reference)   1.39 (0.57-3.39)  1.52 (0.67-3.46)  0.86 (0.24-3.05)  2.01 (0.85-4.78) | -  0.475  0.314  0.816  0.113 |
| Mean Number of HLA-mismatches | 1.23 (0.98-1.54) | 0.075 |
| NODAT | 1.16 (0.36-3.76) | 0.803 |
| Previous transplant | 1.06 (0.51-2.23) | 0.879 |
| Donor Type  Cadaveric  Live | 1.0 (reference)  1.27 (0.70-2.32) | -  0.433 |
| Donor Age (years) | 1.04 (1.02-1.07) | 0.002 |
| Male Donor | 1.12 (0.59-2.14) | 0.736 |
| Donor Race  White  Asian  Black  Other | 1. (reference)   1.00 (1.00-1.00)  1.14 (0.45-2.91)  1.00 (1.00-1.00) | -  0.977  0.779  0.989 |
| Donor BMI (kg/m^2^) | 1.05 (0.99-1.10) | 0.097 |
| CMV Serostatus Combinations  D-/R-  D-/R+  D+/R-  D+/R+ | 1. (reference)   1.03 (0.43-2.48)  0.87 (0.33-2.30)  1.30 (0.59-2.87) | -  0.942  0.785  0.517 |
| CMV D+ | 1.25 (0.78-2.31) | 0.472 |
| CMV R+ | 1.11 (0.61-2.03) | 0.725 |
| Acute rejection in first year | 1.35 (0.60-3.03) | 0.475 |
| Delayed graft function | 2.01 (1.10-3.67) | 0.024 |
| eGFR (ml/min/1.73m^2^)  3 months  12 months  27 months | 0.98 (0.97-1.00)  0.98 (0.96-1.00)  0.97 (0.95-0.99) | 0.045  0.028  0.001 |
| RD >0.8 | 1.22 (0.60-2.48) | 0.578 |

BMI, body mass index; CMV, cytomegalovirus; CI, confidence intervals; D-, donor CMV seronegative, D+, donor CMV seropositive; eGFR, estimated glomerular filtration rate; IMD, index of multiple deprivation; NODAT, new onset diabetes after transplantation; R-, recipient CMV seronegative; R+, recipient CMV seropositive; RD >0.8, greater than 0.8 probability of an episode of rapid deterioration; HR, hazards ratio.

**Supplementary Table 7.** Univariable associations of probability greater than 0.7 or 0.9 for rapid deterioration with all-cause mortality. There were no significant associations consistent with earlier findings with using a cut-off of 0.8.

|  | **Unadjusted HR (95% CI)** | **P-value** |
| --- | --- | --- |
| RD >0.7 | 1.29 (0.65-2.56) | 0.469 |
| RD >0.9 | 1.36 (0.67-2.75) | 0.399 |

RD >0.7, greater than 0.7 probability of an episode of rapid deterioration; RD >0.9, greater than 0.9 probability of an episode of rapid deterioration; HR, hazards ratio.
